# Supplementary material for: Who behaves more pro-environmental in the national parks: A comparison of the tourist and the hiker
Source: PLoS One. 2023 Jun 23;18(6):e0287227. doi: 10.1371/journal.pone.0287227 (PMC10289389; doi:10.1371/journal.pone.0287227)
Supplement: Attachment — Submitted filename: OB-Reviews PLOSONE.docx [file pone.0287227.s001.docx]

Thank you for allowing me to review this manuscript titled “Who take more pro-environmental behavior in national park: Compare the tourist and the hiker”. The paper aims to use Theory of planned behavior and Norm Activation model to explore the intention of tourists’ and hikers' pro-environmental behavior. Overall, the idea is great and provides new knowledge. However, these critical suggestions and corrections are made for the authors to improve the manuscript for possible publication consideration.

**Topic**

The topic, “Who take more pro-environmental behavior in the national park: Compare the tourist and the hiker” is not scientifically clear. I suggest authors rephrase it and check grammar. However, this is suggested to the reviewer for authors. “Who behaves more pro-environmental at the national park: A comparison of the tourist and the hiker

**Abstract.**

1. Authors write “The intention of people’s pro-environmental behavior (PEB) directly affects the sustainable development of national park, but less studies do the compare research of tourist and hiker’ This opening statement is not clear to gain readers’ interest. “What national park?

2. This statement is not clear “The results demonstrate that the TPB model and the NAM model were accept of tourists’ and hikers’ pro-environmental behavior in national park, hikers’ attitude, awareness of consequences, ascription of responsibility were significantly different from the tourist”
3. Authors should provide a sentence of policy implication in the last part of abstract
4. Authors should define “ERB” in its first appearance
5. Abstract has a serious language problem

**Introduction**

1. “…Therefore, given the unique characteristics of mountainous areas, increasing PEB of tourists has emerged as a crucial problem that requires immediate resolution” Authors must provide the unique characteristics of the mountainous areas they talk about.
2. This statement “China is an emerging and vast tourism destination, more than 67% of China's land area is made up of mountainous regions [13], as scenic mountain area attracts large numbers of tourists and hikers, the problem of environmental damage in these areas is getting worse which is also facing various environmental issues” is too long and must be broken down and made clearer.
3. People believed to walk in mountainous areas with different altitudes, which can bring many benefits to participants. What happened today? They don’t believe anymore? Please make statement clear.
4. “As of July 2022, China has a total of 5 National Parks: Sanjiangyuan, Panda, Northeast Tiger and Leopard, Hainan Tropical Rainforest, Wuyishan National Parks.” Please cite a source.
5. What do authors mean by “The management of national parks must be sustainable?
6. Authors claim to do comparative research of a tourist and hiker but only talks about the hikers. Are hikers not tourists? What is the difference between a tourist and a hiker?
7. Authors must highlight clearly the theoretical and practical contribution of the study in the introduction
8. Authors must explain in the introduction what actions they consider pro-environmental at national parks

**Literature review**

1. Section 2 should be Literature review and hypotheses development not “Materials and Methods”
2. Literature review is well written
3. Provide a conceptual framework diagram showing the various association you intend to find to increase readability

**Materials and Methods**

1. Authors can change Section 3 to “Data, materials, and Methods
2. Make this statement clearer ‘Wuyishan National Park is a world natural and cultural heritage recognized by United Nations Educational, Scientific and Cultural Organization (UNESCO), there are only four places in China”
3. In 2022, more than one million tourists “**come**” (came) to Wuyishan National Park for visit.
4. Section 3.2, Based on the language problems in the manuscript, I believe there were misunderstanding issues in the questionnaire designs. Having three professors translate questionnaire. Did the professors check the language for this manuscript as well?
5. Data collection method is well written
6. Was data collected for only Chinese citizens or foreigners included? Please explain

**Results.**

1. Provide table for demographics of both respondent category
2. Measurement model should be in Section 3 and only the results p[resented in section 4
3. Table 2: As suspected there are grammatical issues with the questionnaire items. Example: “I think do PEB in national park is wise” is not clear and can affect respondents response”
4. Hypotheses test results has not been clearly reported.

**Discussion, Conclusion and limitations**

1. Conclusion should be the last section.
2. Avoid citations in policy implication and conclusions
3. Policy implication is scanty done and need improvement. It should focus on what must be done in real life based on the findings from this study
4. There are several limitations that call for additional research, despite the fact that this work offers a fresh viewpoint on the investigation of hikers' **electroreceptor bands…** What does this mean?

**General comments**

1. The manuscript has a serious language problem. There is grammatical error in almost every line which makes it difficult to understand what authors are putting across. Authors need to use a professional language editing tool to significantly improve it
2. Provide line numbers for easy identification and referencing
3. The full meaning of NAM is Norm activation Model, so authors cannot write NAM model which makes it a repetition of “model”
4. For this paper to be considered, it requires a serious improvement especially the language
