# Supplementary material for: Who behaves more pro-environmental in the national parks: A comparison of the tourist and the hiker
Source: PLoS One. 2023 Jun 23;18(6):e0287227. doi: 10.1371/journal.pone.0287227 (PMC10289389; doi:10.1371/journal.pone.0287227)
Supplement: Attachment — Submitted filename: OB-Reviews 2 PLOSONE.docx [file pone.0287227.s003.docx]

Thank you for allowing me to review another round of this manuscript. The authors have improved the revised manuscript according to the comments, but it is still not up to the standard the reviewer wants it. However, the paper may be accepted by the editor after these few minor corrections have been done. Editor does not need to return to me again for checking. You can see to it that they are done and then accept.

1. Check papers published in the journal for proper outline and structure of manuscript.
2. Line 39-41 **“People believe to walk in mountainous areas with different altitudes, which can bring many benefits to” is still not clear**. This is a suggestion.

“People believe walking in mountainous areas with different altitudes can bring many benefits to them”

OR

“It is believed that walking in mountainous areas with different altitudes, can bring many benefits to the participants.”

3. Errror: Traditional visitors have been used as research subjects in the majority of previous studies, but hikers’ present unique characteristics….
4. Authors must number sections and subsections
1. Introduction
2. Literature and hypotheses
3. Methods
4. Etc
