# Supplementary material for: Who behaves more pro-environmental in the national parks: A comparison of the tourist and the hiker
Source: PLoS One. 2023 Jun 23;18(6):e0287227. doi: 10.1371/journal.pone.0287227 (PMC10289389; doi:10.1371/journal.pone.0287227)
Supplement: Attachment — Submitted filename: Response to Reviewers.docx [file pone.0287227.s004.docx]

Response to Reviewer 1 Comments

We would first like to thank you for all the constructive and critical comments on our paper. When revising this paper, we would like to assure you that we have seriously considered all comments and diligently addressed all concerns as thoroughly as possible. Regarding your comments, we have addressed them in our revision as follows.

**Point 1:** Check papers published in the journal for proper outline and structure of manuscript

Response 1: Thank you for your comment, we download the papers published in the Plos one for proper outline and structure of manuscript.

**Point 2:** Line 39-41 “People believe to walk in mountainous areas with different altitudes, which can bring many benefits to” is still not clear. This is a suggestion.

“People believe walking in mountainous areas with different altitudes can bring many benefits to them”

OR

“It is believed that walking in mountainous areas with different altitudes, can bring many benefits to the participants.”

Response 2: we used “It is believed that walking in mountainous areas with different altitudes, can bring many benefits to the participants.” Replace the “People believe to walk in mountainous areas with different altitudes, which can bring many benefits to”

**Point 3:** Errror: Traditional visitors have been used as research subjects in the majority of previous studies, but hikers’ present unique characteristics….

Response 3: we rewrite it. “Traditional visitors have been used as research subjects in the majority of previous studies, but hikers’ present unique characteristic”

**Point 4:**	Authors must number sections and subsections

1.	Introduction

2.	Literature and hypotheses

3.	Methods

4.	Etc

Response 4: we download the paper published in the Plos one, and found that it do not need to add the number sections and subsections.

Response to Reviewer 2 Comments

We would first like to thank you for all the constructive and critical comments on our paper. When revising this paper, we would like to assure you that we have seriously considered all comments and diligently addressed all concerns as thoroughly as possible. Regarding your comments, we have addressed them in our revision as follows.

**Point 1:** The authors responsed and revised the question very well. However, I suggest the authors combine the figure 1 and figure 2 as a whole conceptual model.

**Response 1:** we combine the figure 1 and figure 2 as a whole conceptual model. See the figure 1 Conceptual model.
